# Study on crystal growth of Ge/Si quantum dots at different Ge deposition by using magnetron sputtering technique

**DOI:** 10.1038/s41598-023-34284-8

**Published:** 2023-05-09

**Authors:** Qijiang Shu, Pengru Huang, Fuhua Yang, Linjing Yang, Lei Chen

**Affiliations:** 1 Institute of Information, Yunnan University of Chinese Medicine, Kunming, 650500 China; 2 School of Material Science and Engineering, Guangxi Key Laboratory of Information Materials and Guangxi Collaborative Innovation Center of Structure and Property for New Energy and Materials, Guilin University of Electronic Technology, Guilin, 541004 China; 3 College of Chinese Materia Medica, Yunnan University of Chinese Medicine, Kunming, 650500 China; 4 Faculty of Narcotics Control, Yunnan Police College, Kunming, 650223 China

**Keywords:** Nanoscience and technology, Nanoscale materials

## Abstract

We investigated the growth and evolution of Si-based Ge quantum dots (Ge/Si QDs) under low Ge deposition (1.2–4.4 nm thick) using magnetron sputtering. The morphology and structure of QDs were analyzed with the help of an atomic force microscope (AFM), scanning electron microscope, transmission electron microscope, Raman, surface energy theory and dynamics theory, the photoelectric properties of QDs were characterized by photoluminescence (PL) spectra. The results showed that the growth mechanism of QDs conformed to Stranski–Krastanow mode, but the typical thickness of the wetting layer was nearly three times higher than those derived from conventional technologies such as molecular beam epitaxy, chemical vapor deposition, solid phase epitaxy and so on. Meanwhile, the shape evolution of QDs was very different from existing reports. The specific internal causes of these novel phenomena were analyzed and confirmed and reported in this paper. In addition, the AFM, Raman, and PL tests all indicated that the QDs grown when 3.4 nm Ge was deposited have the most excellent morphology, structure, and optoelectronic performance. Our work lays a foundation for further exploration of the controllable growth of QDs at high deposition rates, which is a new way to realize the industrialization of QDs used for future devices.

## Introduction

Owing to many advantages, such as compatibility with Si-based technology, long carrier lifetime, δ-shaped density of states, strong response to near-infrared and mid-infrared bands, etc., Ge quantum dots (QDs) have shown a wide range of applications in optoelectronic devices^1–8^ and attracted a lot of research work^9–11^. Over the past 30 years, Ge QDs have been grown mainly by materials equipment with low deposition rates (~ 0.01–0.04 Å/s), such as molecular beam epitaxy (MBE)^12–14^, chemical vapor deposition (CVD)^15–18^, and solid phase epitaxy (SPE)^19^, their corresponding complete technical system has been formed. In recent years, the idea of exploring the growth of Ge QDs has been further broadened, some work using typical physical vapor deposition (PVD) techniques such as ion beam sputtering^20^, high-vacuum evaporation^21^, e-gun deposition^22,23^ to prepare Ge QDs has been reported.

In the past, researchers have been under the prejudice that excessively fast deposition by magnetron sputtering tends to introduce dislocation defects that affect the crystallization of films and fail to grow the desired QDs. However, once the sputtering parameters are adjusted to the suitable conditions for surface migration of sputtered atoms to form nano-islands, assisted by the interaction of dynamics and thermodynamics, magnetron sputtering is expected to become a more efficient, cheaper, and simpler method for preparing QDs than the conventional technologies. These attractive advantages have led to a lot of research work. Abd Rahim et al. fabricated low-density Ge QDs with a size of 2.65–3.5 nm by performing rapid annealing (400–800 °C) of amorphous Ge films sputtered at room temperature on Si surfaces^24^. Samavati et al.^25^ studied the effects of various parameters of RF magnetron sputtering, including substrate temperature, deposition time, Ar flow rate, RF power, working pressure, annealing temperature, and annealing time, on the structure and properties of Si-based Ge quantum dots (Ge/Si QDs). Khelidj et al. used magnetron sputtering to grow Ge_1−*x*_Sn_*x*_ films with potential for CMOS (complementary-metal-oxide-semiconductor) applications and analyzed the nanostructure and electrical properties of the Ge–Sn alloys in these films^26^. In addition, the work on the formation of multi-layer Ge QDs in Ge/SiO_2_ or Ge/Si bi-layers with multiple repetition periods grown by magnetron sputtering has also been reported^27,28^. However, even so, due to the late start of magnetron sputtering in the preparation of QDs, there are still experimental and theoretical blank areas in the growth technics and mechanism. Particularly worth mentioning is that in the reported related research, the formation of QDs was mostly achieved by sputtering thicker (250 nm^24^, 30–66 nm^25^, 100 nm^26^, etc.) Ge films, while excessive deposition thickness means more dislocation defects, which not only reduces the photoelectric properties of films but also adversely affects and interferes structure and performance of surface QDs, which poses a challenge to the device application of QDs. Therefore, it is very meaningful to further study the growth of Ge QDs, especially the Ge/Si QDs with low Ge deposition, by using magnetron sputtering.

In this paper, a deposition rate that was beneficial to the migration and nucleation of sputtered atoms was obtained by adjusting the sputtering parameters, and the corresponding crystallization temperature of the deposited film and the thickness of the Ge layer required for the formation of Ge QD were explored. Referring to the thickness of the Ge layer (~ 1–2 nm) in the process of growing Ge/Si QDs with MBE and CVD, the evolution mechanism of Ge/Si QDs by direct current (DC) magnetron sputtering under a small deposition amount (several nanometers) was systematically studied. Our work establishes the basis for the future use of magnetron sputtering to efficiently grow Ge/Si QDs for optoelectronic devices.

## Methods

### Sample preparation

All the samples in this paper were grown in a magnetron sputtering equipment with a background pressure of 3.0 × 10^–4^ Pa. Single-side polished Si (100) with 500 μm (n-type conductivity) was used as the substrate to be cleaned with Shiraki standard method, and then rinsed with HF acid solution with a concentration of 2.5% for 60 s to remove some passivated H atoms and the natural oxide layer on the substrate surface. The targets used for sputtering Si and Ge were the cylindrical targets with a high purity of 99.99%, and the working gas was argon with a purity of 99.999% and in continuous flow mode with a set flow rate of 5 sccm. The sputtering power and pressure were adjusted to the minimum allowable value of the equipment to provide sufficient surface migration time for sputtered atoms to promote film crystallization and atomic nucleation. Specifically, Si was sputtered by RF (radio frequency) mode, the sputtering power and pressure were 45 W and 0.4 Pa, Ge was sputtered by DC (direct current) mode, and the sputtering power and pressure were 35 W and 0.2 Pa respectively.

In order to quantify the deposition rate of Si and Ge and determine the crystallization temperature of Si and Ge, Si was sputter-deposited for 7200 s on the substrate with a temperature of 20 °C (room temperature), 200 °C, 400 °C, 600 °C, 700 °C, and 800 °C to prepare samples Si_20_, Si_200_, Si_400_, Si_600_, Si_700_, and Si_800_, Ge was sputter-deposited for 3600 s on the substrate with a temperature of 20 °C, 100 °C, 300 °C, 500 °C, 600 °C, and 700 °C to prepare samples Ge_20_, Ge_100_, Ge_300_, Ge_500_, Ge_600_, and Ge_700_ respectively. The deposition rate was obtained by calculating the ratio of film thickness to growth time, and the crystallization temperature was determined by Raman measurement and analysis.

Based on the research results of deposition rates and crystallization temperatures of Si and Ge, a 50 nm Si buffer layer was first deposited on the substrate at 750 °C and annealed for 30 min to fabricate a crystalline film to cover the substrate-surface impurities that were difficult to be removed by cleaning, so as to obtain a flat Si surface suitable for the growth of Ge QDs. Then, the single-layer QD samples Ge_1.2_, Ge_1.6_, Ge_2.0_, Ge_2.4_, Ge_3.4_, and Ge_4.4_ were obtained by sputtering a Ge film with the thickness of 1.2 nm, 1.6 nm, 2.0 nm, 2.4 nm, 3.4 nm, and 4.4 nm respectively on the Si buffer layer at 550 °C. To minimize the influence of the atmosphere on the samples, all samples taken out of the sputtering chamber were put into bags filled with nitrogen gas with a purity of 99.999% to wait for measurement.

### Material characterization

The morphology, structure, and optoelectronic properties of QDs were characterized with atomic force microscopy (AFM), scanning electron microscope (SEM), transmission electron microscope (TEM), Raman, and photoluminescence (PL). All the AFM measurements were performed on the same instrument (SPA-400SPM), in which an ultra-fine tip (SSS-SEIHR) with a small radius of curvature was employed to improve the accuracy by running tapping mode, the AFM, SEM, TEM, and Raman tests were all performed at room temperature, while the PL tests were carried out with the excited laser wavelength of 488 nm, power of 0.8 mW and the signal recording temperature of 17 K, respectively.

## Results and discussion

### Deposition rates and crystallization temperatures

**Figure 1:**
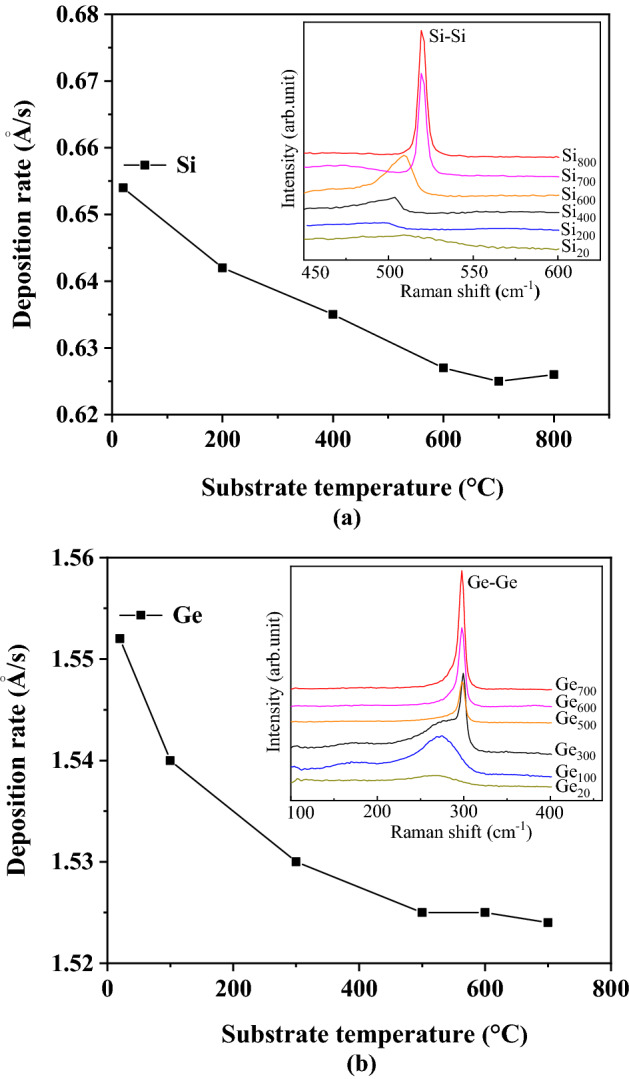
The deposition rates of the films at different temperatures and the Raman spectra of these films, (**a**) and (**b**) correspond to the measurement results of the deposited Si and Ge films, respectively.

In the experiments, the deposition rate of Ge or Si was defined as the ratio of the thickness of the Ge or Si films to their corresponding growth time, where the thickness of these films was measured by a DektakXT-type step profiler with a repeatability accuracy of less than 4 Å and a vertical resolution of 1 Å. As we all know, the number of sputtered atoms is constant under the same sputtering power, pressure, and time, while these sputtered atoms deposited on a substrate will form films with different quality (crystallinity, compactness, adhesion, and thickness) due to the different substrate temperatures, which inevitably results in different measurements for deposition rates. Researching the correlation between deposition rate and substrate temperature is the basis for quantitatively fabricating QD samples at different temperatures. Therefore, an experiment was designed to deposit Si and Ge, the results are shown in Tables 1, 2. The variation curves of the film deposition rates calculated from these film samples grown at different temperatures are shown in Fig. 1, the Raman spectra of these films are also shown in the upper right of the rate curves in the form of insets for crystallographic analysis.

**Table 1:** Growth of Si film at different substrate temperatures.

Sample	Si_20_	Si_200_	Si_400_	Si_600_	Si_700_	Si_800_
Substrate temperature/°C	20	200	400	600	700	800
Growth time/S	7200	7200	7200	7200	7200	7200
Film thickness/Å	4712	4622	4572	4515	4500	4507
Deposition rate/Å/s	0.654	0.642	0.635	0.627	0.625	0.626

**Table 2:** Growth of Ge film at different substrate temperatures.

Sample	Ge_20_	Ge_100_	Ge_300_	Ge_500_	Ge_600_	Ge_700_
Substrate temperature/°C	20	100	300	500	600	700
Growth time/S	3600	3600	3600	3600	3600	3600
Film thickness/Å	5587	5544	5508	5490	5490	5486
Deposition rate/Å/s	1.552	1.540	1.530	1.525	1.525	1.524

As can be seen from Tables 1, 2, and Fig. 1, at the same growth time, the thickness of Si and Ge films (deposition rate) decreases gradually with increasing temperature and finally tends to be constant. Such a result is easily explained: the atoms sputtered at low temperatures do not get enough diffusion momentum, and coupled with the high deposition rate of magnetron sputtering that does not provide enough migration time for the sputtered atoms (they are immediately covered by the next batch of sputtered atoms), the nuclei formed by atomic coalescence during the film growth process hardly crystallize and grow up, they simply stack, forming an overall fluffy film with a large thickness. The increased temperatures effectively promote the diffusion and migration of sputtered atoms, prompting the condensation nuclei to crystallize, grow, and then interconnect with each other, the film morphology develops into a mixed crystal state or even close to a single crystal state, and the film compactness is greatly enhanced and the thickness is thus reduced. Apparently, the increase in crystallization rate will tend to be constant after the temperature rises to a certain value, and the film thickness therefore no longer changes.

In the inset of Fig. 1a, the Raman peak (Si–Si peak) of Si film approximately at 500–520 cm^−1^ increases and gradually shifts to 520 cm^−1^ with the substrate temperature. The Si–Si peak with a large peak strength and a small FWHM (full width at half maximum) indicates good crystallinity of Si when the temperature is higher than 700 °C. A similar phenomenon can be observed in Fig. 1b, that is, the Raman peak (Ge–Ge peak) of Ge film gradually shifts to 300 cm^−1^ with the increase in temperature. And the temperature higher than or equal to 500 °C can decrease or eliminate the amorphous phase of Ge to improve the crystalline fraction of the sample. These results are consistent with the analysis and inference obtained from the variation curves of film deposition rates. Based on the above results, to ensure the crystallinity of Ge/Si QDs as much as possible and avoid large-scale Si-Ge atoms intermixing caused by excessive temperature, in the growth of QDs, the growth temperatures of Si buffer layer and Ge film were set to 750 °C and 550 °C, the corresponding deposition rates were considered to be 0.625 Å/s and 1.525 Å/s according to Tables 1, 2, respectively.

### Morphological analysis of Ge/Si QDs

**Figure 2:**
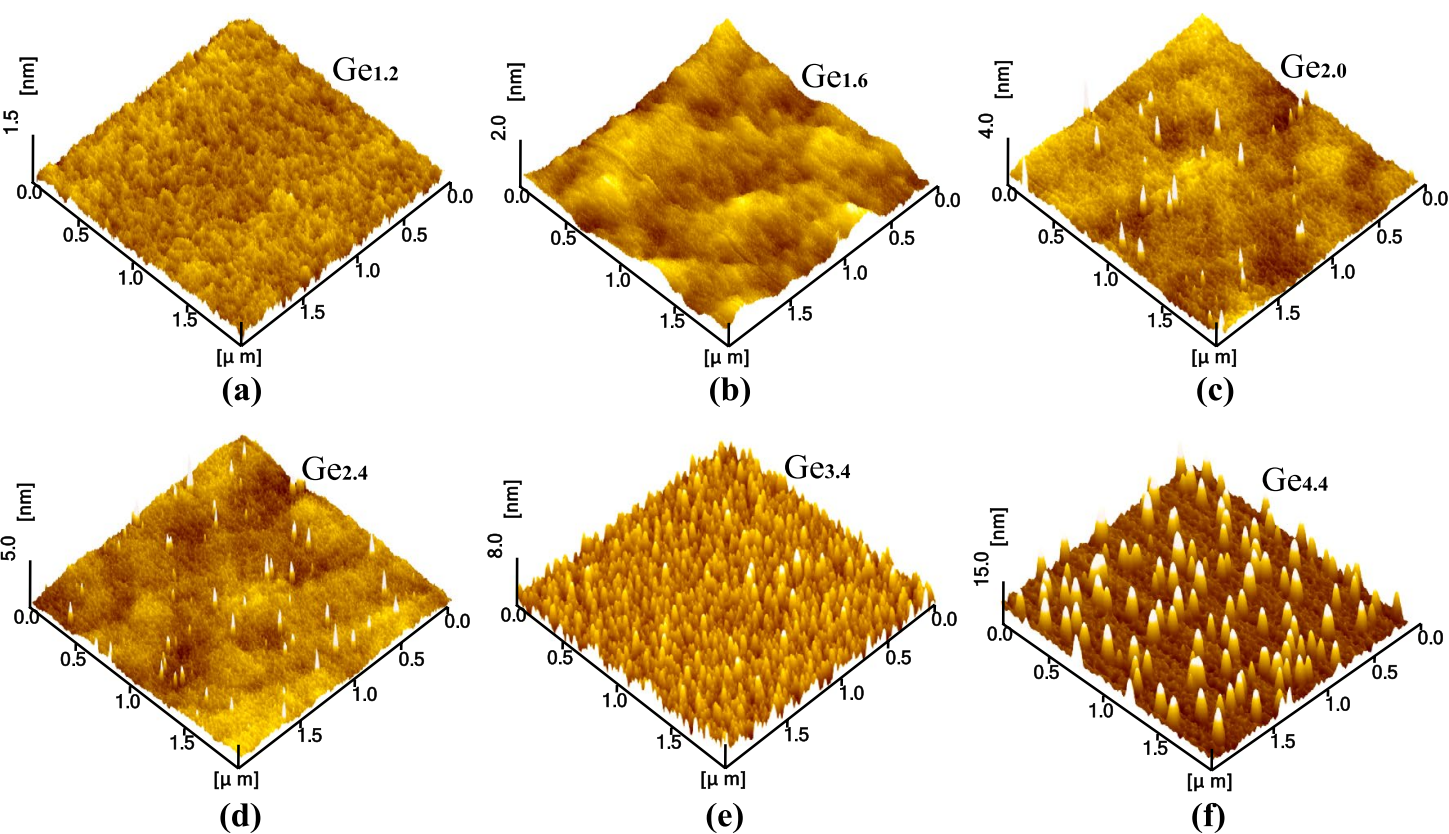
Surface morphologies of samples with different Ge deposition thicknesses (1.2 nm, 1.6 nm, 2.0 nm, 2.4 nm, 3.4 nm, and 4.4 nm) measured by AFM.

**Figure 3:**
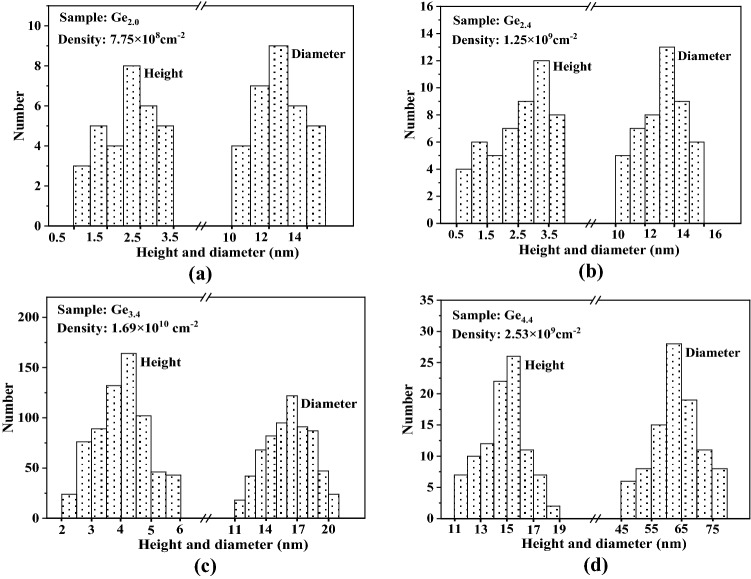
The statistical histograms of QD size of samples Ge_2.0_, Ge_2.4_, Ge_3.4_, and Ge_4.4_ (**a**, **b**, **c**, and **d**). The QDs in the area of the same size as each sample were counted to make a histogram to exhibit the different distribution intervals of QD height and QD diameter (as shown in abscissa) and the corresponding number of QDs in this numerical interval (as shown in ordinate). The corresponding QD density is also marked in each histogram (upper left corner).

The AFM photos of three-dimensional QDs in samples (Ge_2.0_, Ge_2.4_, Ge_3.4_, and Ge_4.4_) are shown in Fig. 2. When the thickness of Ge film increases from 1.2 to 1.6 nm (Ge_1.2_ and Ge_1.6_), no island-like structures are found on the surface of the film. When the deposition amount of Ge is continuously increased to a thickness higher than 2.0 nm, the surface of each sample exhibits QDs with different morphologies. The QD morphology information (density, height distribution, and diameter distribution) of samples Ge_2.0_, Ge_2.4_, Ge_3.4_, and Ge_4.4_ are shown in Fig. 3. Combined with the data of Fig. 2 and Fig. 3, as the deposition thickness of Ge film varies from 2.0 nm to 2.4 nm and 3.4 nm, an associated rise in density and size of QDs, especially when the film thickness is 3.4 nm, and the density reaches 1.69 $\times$ 10^10^ cm^−2^, with a height of 2–6 nm and a diameter of 11–22 nm. More quantitatively, the diameter change of QDs is relatively small, and the density and height are the main increases. Compared with the sample Ge_3.4_, when the Ge deposition thickness is further increased to 4.4 nm, the height and diameter of QDs dramatically change to 11–19 nm and 45–80 nm respectively, while the dot density drops sharply to only 2.53 $\times$ 10^9^ cm^−2^. The above statistical parameters of the QDs are similar to some of the results that have been reported for self-organized QDs, for example, the Ge/Si QDs prepared by Qayyum et al.^29^ using a line-focused pulsed laser beam scanning a pre-deposited Ge film on Si substrate to have a height of 2.9 nm, a diameter of 25 nm, and a density of 6.0 $\times$ 10^10^ cm^−2^. And the Ge QDs grown on the Si buffer layer by Brehm et al.^30^ using the MBE technique showed two typical structures, in which the densities of pyramid-shaped and dome-shaped QDs were 3.8 $\times$ 10^9^ cm^−2^ and 5.7 $\times$ 10^9^ cm^−2^, with corresponding average heights of 4 nm and 11 nm and average diameters of 50 nm and 80 nm, respectively.

The morphological evolution of the above QDs can be analyzed by the Stranski–Krastanow (SK) growth mode. During the initial stage of sputtering Ge (Ge_1.2_ and Ge_1.6_), due to the lattice mismatch between Ge and Si, a large amount of compressive strain is accumulated into the grown Ge film, and such strain can be expressed as^31^:

1 $$ E_{\varepsilon } = 2\mu_{e} \frac{1 + v}{{1 - v}}hf^{2} $$

where ${\mu }_{e}$ and $v$ represent the shear strain and Poisson ratio of Ge film respectively, *h* is the thickness of Ge film, and *f* is the lattice mismatch. From Eq. (1), the strain increases with the film thickness, during which some of the strain relaxes to drive the film nucleation and 2D (two-dimensional) growth until the film thickness exceeds a critical value, the strain beyond its threshold is released in the form of forming nanoislands, that is, the deposited Ge starts its 3D (three-dimensional) growth^32,33^ (Ge_2.0_). Continuing to raise the number of deposited atoms, which move, collide, and condense on the film surface, not only promotes the rapid growth of those already formed nuclei into QDs, but also enhances the probability of forming new nuclei and developing them into QDs, thus increasing the size and density of the QDs (Ge_3.4_). The elevated density reduces the spacing between the QDs, making the elastic spaces between the QDs overlap each other, which makes these QDs compete when gathering the atoms around them. When further sputtering atoms are added, the QDs with low chemical potential are easy to agglomerate atoms and grow preferentially until they overlap with the surrounding islets and annex them to form super-large islands. As a result, the size of QDs increases rapidly and the density drops sharply, as shown in Ge_4.4_, exhibiting a typical Ostwald ripening process.

**Figure 4:**
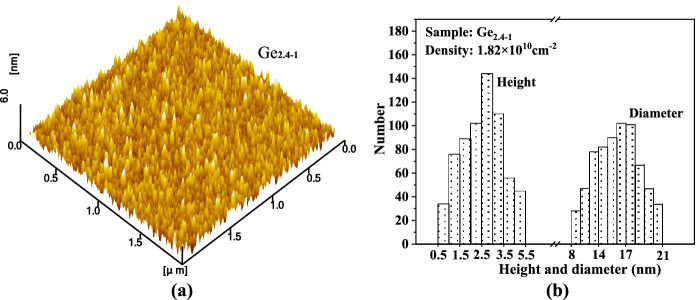
AFM image of the control sample Ge_2.4−1_ (**a**) and the statistical histogram of QD size of sample Ge_2.4−1_ (**b**). The corresponding QD density is also marked in a histogram (upper left corner).

It must be noted that, following SK mode, the critical thickness of the deposited Ge is between 1.6 and 2.0 nm (i.e. 11–14 ML) from Fig. 2, which is much higher than the typical values of 3 ML-6 ML obtained by conventional methods such as MBE, CVD, SPE and so on^12,13,15,19^. The corresponding QD density of sample Ge_2.0_ (~ 10^8^ cm^−2^) is also much lower than the reported values (~ 10^9^–10^10^ cm^−2^). As we have elaborated in our discussion of the results in Fig. 1, high deposition rates can lead to insufficient migration of sputtering atoms, the surface flatness and compactness of the grown Ge film are not high (as shown in Fig. 2a–c), the film is not a standard monolithic single crystal but is composed of regional polycrystalline structures. This means that there is a large difference between the lattice mismatch between Ge and Si grown in the sample and the theoretical value of 4.2% calculated from their single crystals. Focusing on Eq. (1), a strong possibility is that this lattice mismatch arising from the actual growth process is considerably lower than the theoretical value, and therefore a larger film thickness is required to accumulate strain energy to reach the corresponding critical threshold. At the same time, insufficient migration also reduces the probability that atoms meet each other to form nuclei and evolve into QDs, so the QD density is low. An easy way to verify the above inference is to increase the substrate temperature to improve the atomic migration ability of the substrate surface while maintaining other same sputtering parameters to obtain a control sample. Thus, sample Ge_2.4−1_ was prepared at 650 °C using the same sputtering parameters as Ge_2.4_. The AFM measurement of sample Ge_2.4−1_ and the statistical information of its QD morphology are shown in Fig. 4.

Compared with sample Ge_2.4_ (Figs. 3d, 4b), the density of QDs in sample Ge_2.4−1_ is increased by more than 10 times, and the corresponding QD size and size distribution range are significantly enlarged. Based on the fact that the Ge deposition in these two samples is equal, it can be inferred that the process of Ge growth from 2D layers to 3D islands in Ge_2.4−1_ occurs earlier than that in Ge_2.4_ (the critical thickness becomes thinner) because more Ge atoms in Ge_2.4−1_ are used to fabricate QDs. Such a result indirectly supports the reason for the formation of the Ge critical thickness in Fig. 2, and also inspires another interesting research topic, namely the effect of different substrate temperatures on the critical thickness and QD morphology, which is not the focus of this paper and will be discussed in our other work.

### Shape evolution of Ge/Si QDs

**Figure 5:**
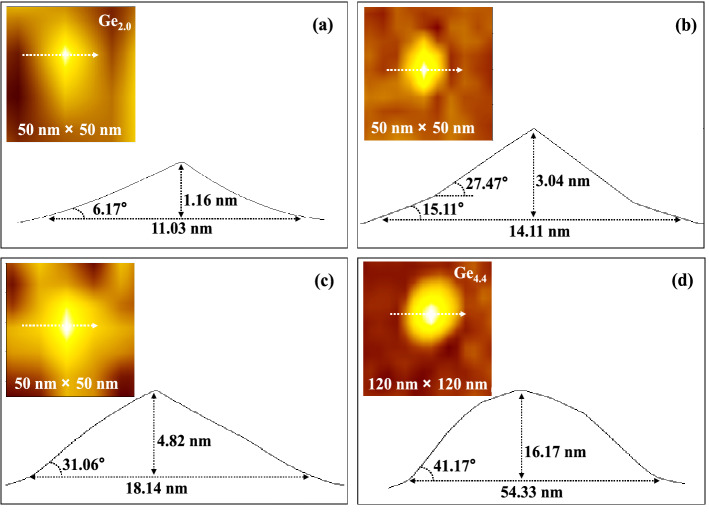
Top-view photos of typical single QD in samples Ge_2.0_, Ge_2.4_, Ge_3.4_, and Ge_4.4_ (as shown in the upper left corner of (**a**), (**b**), (**c**), and (**d**) respectively) and the corresponding profile morphology scanning along the white arrow with the thick dotted line across the single QD (shown as the bottom of (**a**), (**b**), (**c**) and (**d**) respectively).

The top-view photos of representative individual QD in each sample and the corresponding contour morphology and size parameters scanned along a specific direction are shown in Fig. 5. Referring to the reported experimental fact that different crystal planes of the constructed QDs exhibit different tilt angles relative to the ground plane^34–37^, as shown in Fig. 5, the initial shape of the QD is a pyramid constructed from a standard single crystal plane, and its aspect ratio is close to 0.1 (sample Ge_2.0_). In sample Ge_2.4_, the aspect ratio of the QD goes up close to 0.25, and the corresponding pyramid shape is constructed from multiple crystal planes (sample Ge_2.4_). From Ge_2.4_ to Ge_3.4_, the height of QD increases significantly and the diameter changes less, the bottom contact angle of QD increases faster than its top side angle, the QD gradually forms the shape with cylindrical bottom and sharp top, and the QD top still maintains the pyramid shape with the aspect ratio value close to 0.27 in Ge_3.4_. For the QD in sample Ge_4.4_, its diameter and height increase sharply, the change of contact angle on the QD bottom is slower than that on the top side, there are many crystal planes with small tilt angles (low-index crystal planes) to build the top side of QD to show a dome shape.

The transition of the QD shape from a pyramid to a dome is the result of the constant change of the competition between the surface energy and strain energy of films in self-organized growth. Such a principle has been reported in many kinds of literature. However, it is interesting that the aspect ratio of the pyramid shape shown in our results is very different from the existing reports. For example, Ribeiro^34^ and Ross et al.^35^ employed MBE to prepare Ge/Si QDs, and when the aspect ratio of QDs only exceeded 0.1, their shape began to change from pyramid to dome. Similarly, Zhang^36^ and Yang et al.^37^ used ion beam sputtering fabricated short-small Ge/Si QDs, which transformed into large domes when their aspect ratio rose to about 0.1. To reveal the regulatory effect of the core factors in crystal self-assembled growth on the shape transformation of Ge/Si QDs, we establish a model, as follows:

According to the available literatures^33,38^, the total energy of Ge QD constructed from crystalline planes with the same energy can be expressed as:

2 $$ E_{Ge} = V^{2/3} \alpha^{4/3} - V\alpha $$

And the total energy of a Ge QD mixed with a certain percentage of Si atoms can be expressed as:

3 $$ E_{GeSi} = V^{2/3} \alpha^{4/3} - V\alpha + [1 - \left( {1 - x)^{2} } \right]V\alpha $$

where *V* is the volume of the QD, *α* is the angle between the side of the QD and the growth surface. The $[1-(1-x{)}^{2}]V\alpha$ can be seen as the additional energy term brought to the QD by the mixing of Si atoms, and $[1-(1-x{)}^{2}]$ is the energy term coefficient related to the occupancy ratio *x* of Si atoms within the QD. Considering the generation of dislocation defects in QDs, we imitate the form of the energy term of atomic intermixing in (3) and write the additional energy term brought to the QD by dislocations as *yVα*. The reasonableness of this additional energy term is ensured by an energy term coefficient *y* with high inclusiveness (its specific form is determined by factors such as the type, location, and density of dislocations, and specific actual detection data are required to determine it). Thus, the total energy of a QD can be written as:

4 $$ E_{Q}^{\prime } = V^{2/3} \alpha^{4/3} - V\alpha + \left[ {\left( {1 - x} \right)^{2} } \right]V\alpha + yV\alpha $$

When a pyramid-shaped QD is transformed into a dome-shaped QD, their corresponding energy is equal. From this condition, the critical volume and diameter of QD could be solved and expressed as follows:

5 $$ V_{0}^{\prime } = \left( {\frac{{\alpha_{P}^{4/3} - \alpha_{D}^{4/3} }}{{\alpha_{P} - \alpha_{D} }}} \right)^{3} \cdot \frac{1}{{\left( {1 - 2x + x^{2} - y} \right)^{3} }} $$

6 $$ d_{0}^{\prime } = \frac{{d_{0} }}{{1 - 2x + x^{2} - y}} $$

where ${\alpha }_{P}$ and ${\alpha }_{D}$ are the contact angles of pyramid-shaped and dome-shaped QDs, respectively, ${d}_{0}$ is the critical transition size of the ideal QD (without Si component and dislocations). It is obvious from Eqs. (5) and (6) that ${V}_{0}^{^{\prime}}$ and ${d}_{0}^{^{\prime}}$ are very sensitive to the percentage *x* of Si atoms mixed in the QD and the energy coefficient *y* corresponding to the dislocations, and small variations in both *x* and *y* can produce large changes in the critical value, meaning that the transformation process is advanced or delayed. The numerical variations of *x* and *y* are commonly present in the growth of QDs, and these variations vary considerably among deposition techniques, this explains why the shape evolution of our sputtered QDs differs from other results that have been reported. It can be predicted that even if the same technique is adopted, if the growth parameters (such as sputtering power, sputtering pressure, growth temperature, etc.) are greatly adjusted, the shape evolution of QDs will show different results and laws. Depending on Eqs. (5) and (6), due to the complex nonlinear relationship between the *x*, the *y*, the crystal plane growth orientation and different growth parameters, these results and laws need to be explored by a lot of experiments. The results obtained under the parameters in this paper are the basis for these future studies.

It is worth noting that there is a tip convolution effect when using AFM to determine the topography of QDs, which will overestimate the lateral size of QDs. According to the modified models proposed by Gong et al.^39^ and Sheng et al.^40^ (referred to as Model 1 and Model 2, respectively), the use of the probe with the tip as thin as possible could effectively reduce the error caused by this effect. Therefore, in our work, an ultra-fine tip of type SSS-SEIHR with a small radius of curvature (< 5 nm) was used for AFM measurement. Taking Ge_4.4_ as an example, the statistical average of the diameter of QDs observed by AFM is 63.34 ± 7.88 nm, and the corrected value calculated by bringing this value and probe parameters into Model 1 and Model 2 are both 60.97 nm. The measured and corrected values are very close, and the small difference between them is well within the allowable range of statistical error, which indicates that the measurement accuracy of the AFM in our work is tolerable.

**Figure 6:**
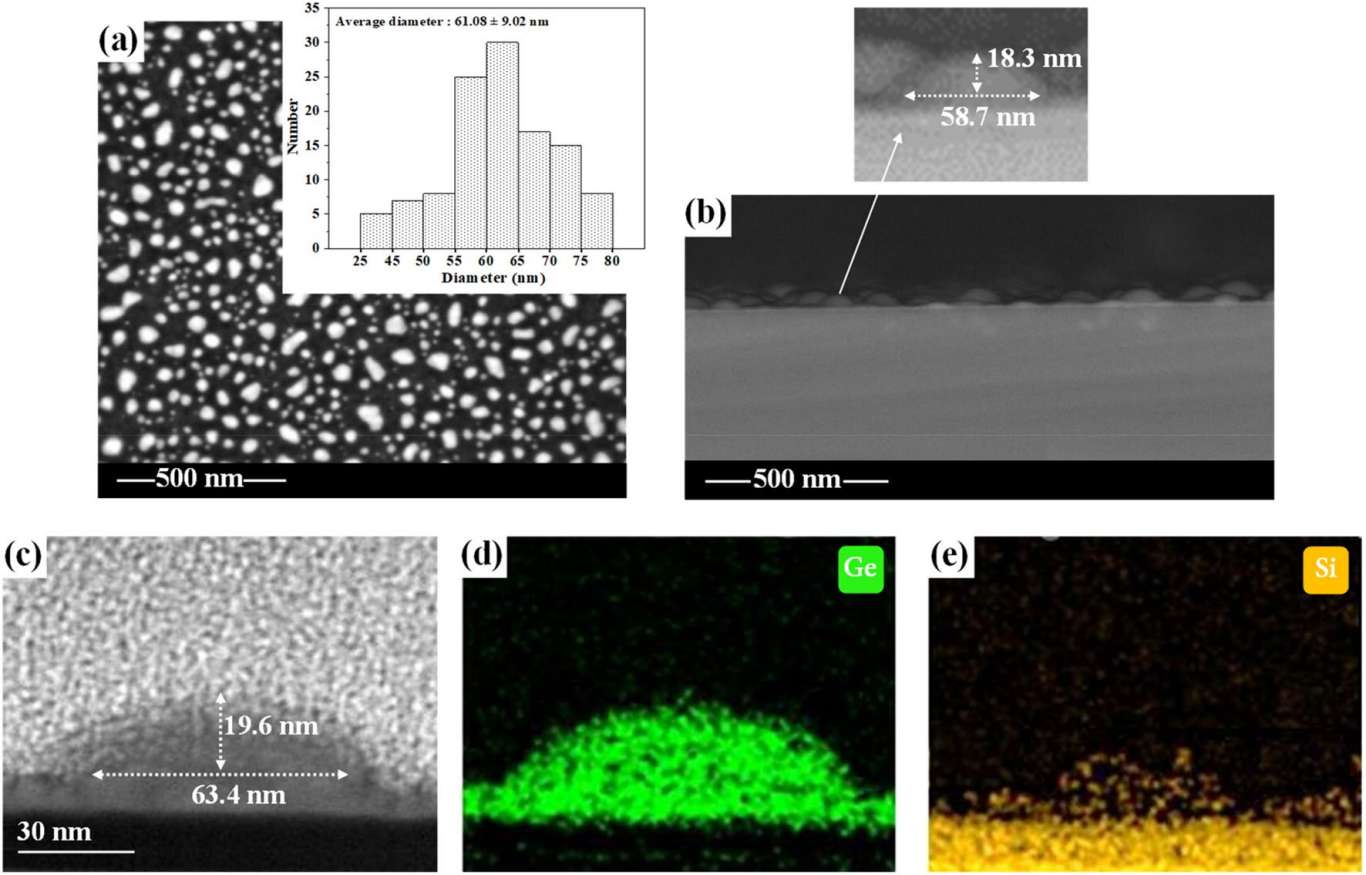
SEM and TEM micrographs of sample Ge_4.4_. (**a**) and (**b**) are the overhead view and sectional view of QDs on the sample surface measured by SEM at a magnification of 200,000, respectively. The inset to the upper right of (**a**) is a histogram of the diameter distribution of these QDs, and the inset above (**b**) is an enlarged view and size of a typical QD in (**b**). (**c**) is a TEM image of a typical single QD in Ge_4.4_, in which the diameter and height of this QD are marked, and the (**d**) and (**e**) reveal respectively the spatial distribution of Ge and Si in this QD measured from TEM elemental mapping.

In addition, we measured sample Ge_4.4_ with SEM and TEM, respectively, the results are shown in Fig. 6. Figure 6a is a plan-view SEM micrograph of the surface QDs on sample Ge_4.4_, and the inset at the upper right of (a) is the statistical histogram of the diameters of these QDs. By comparing the histogram in Fig. 6a with that in Fig. 3d, it can be found that the distribution characteristics of QD diameters shown by the two are very similar. Furthermore, the statistical average value of the QD diameter with a value of 61.08 ± 9.02 nm shown in the histogram in Fig. 6a is also very close to the value of 63.34 ± 7.88 nm measured by AFM. Figure 6b is a cross-sectional SEM image of the surface QDs of sample Ge_4.4_. The inset above (b) shows a partially enlarged picture of a typical QD in (b), where the QD diameter and height are noted. Similarly, Fig. 6c shows a cross-sectional TEM micrograph of a typical single QD in Ge_4.4_. Comparing Fig. 6b–d, the measurement results of the three are quite similar. Figure 6d,e are the spatial distribution pictures of Ge and Si in QD tested by TEM mapping, respectively. The corresponding percentage of the number of Si atoms in the Ge QD in the figure is 11.67% by the EDS-HAADF test. Such a result shows that there is obvious Ge-Si interdiffusion at their contact interface, and the amount of Si diffuses into the Ge layer and Ge QD is significantly more than the amount of Ge immerses into the Si layer. This (asymmetric diffusion) is consistent with the results observed by Tripathi et al.^41^.

### Crystallographic and optical properties of Ge/Si QDs

**Figure 7:**
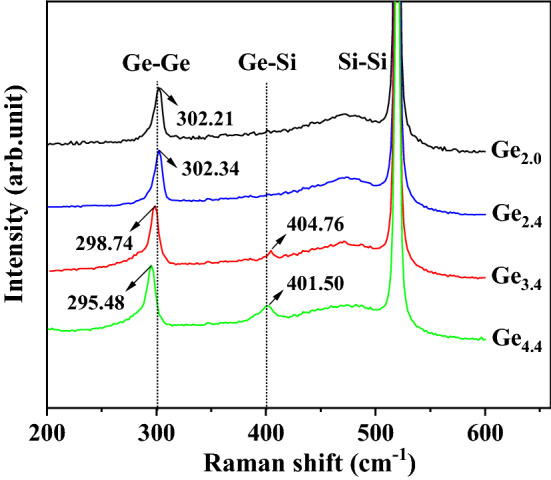
Raman spectra of samples Ge_2.0_, Ge_2.4_, Ge_3.4_, and Ge_4.4_. The text labels in the figure indicate the Raman signals related to the Si layers (Si–Si), Ge–Si interdiffusion (Ge–Si), and Ge/Si QDs (Ge–Ge), and the central positions of these peaks are marked in the figure.

The Raman spectra of the samples Ge_2.0_, Ge_2.4_, Ge_3.4_, and Ge_4.4_ are shown in Fig. 7. As shown in Fig. 7, a clear characteristic peak of Ge–Ge vibrational mode is observed near 300 cm^−1^ in all samples, and the non-perfect symmetry of their peak patterns indicate that the grown Ge films are not perfect single crystals, which is consistent with the previous inference, while the small FWHM and high intensity of these peaks imply that the Ge films are well crystalline, and such results are consistent with the inset of Fig. 1b and the experimental expectation. In addition to showing similar results to the Ge–Ge peaks described above, the Si–Si peaks also show broad amorphous Si signals near 480 cm^−1^, which are similar to the measurement of Si_700_ and different from that of Si_800_ in Fig. 1a, indicating that a small percentage of the amorphous phase is still present in the Si buffer layer grown at 750 °C. These amorphous Si are likely to occur in two processes: First, during the stacking of deposited atoms into films, some atoms form amorphous Si due to insufficient diffusion and migration. Second, there are some impurities and H atoms on the surface of the non-ideal Si substrate that are difficult to remove by cleaning. The deposited silicon atoms first migrate between these obstacles and fill the convex and concave parts of the surface itself, thus forming some amorphous wedges located at the bottom of the film, as observed by Sasaki et al.^42^.

The continuous increase of Ge deposition intensifies the slight interdiffusion between Ge and Si, which could be reflected by the gradual enhancement of the characteristic peak of Ge–Si mixing near 400 cm^−1^ in samples Ge_3.4_ and Ge_4.4_. Such a result is easily explained. The increased deposition of Ge brings about two changes, one is the prolongation of the atomic diffusion coefficient due to the extension of deposition time; the other is that the Ge QDs undergo an Ostwald ripening process in which the large islands annex their surrounding small ones (as discussed in Fig. 2), which leads to a large adjustment of the lattice strain in the islands. This is a process in which relatively more dislocation defects are easily generated, and the chemical potential of the islands is further reduced, which provides more possibilities for the interdiffusion of Ge and Si atoms at the bottom of the islands. Therefore, a relatively strong Raman peak from Ge–Si bond vibration is observed in sample Ge_4.4_ in Fig. 7, which also verifies the measurements shown in Fig. 6d,e. The weak Ge–Si peak in the figure means that the temperature of 550 °C does not provide enough kinetic energy for atoms to migrate across the barrier between the highly crystalline film layers to generate large-scale interdiffusion. These growth parameters and results are significant for growing QDs with high Ge content to exert their properties. In addition, referring back to Eqs. (5) and (6), from the measurement results in Fig. 7, it can be inferred that the shape (chemical potential) evolution of QDs in samples Ge_2.0_ and Ge_2.4_ is almost not affected by the Si content *x*, when the Ge deposition reaches 3.4 nm and 4.4 nm, the change of Si content will lead to a great transformation in the shape of QDs, this conjecture is indeed verified by the results in Fig. 5c,d.

As can be seen from Fig. 7, the Ge–Ge peak of each sample has a different degree of frequency shift compared to the intrinsic peak of Ge bulk material at 300.5 cm^−1^. Many existing reports^14,43,44^ have pointed out that in heteroepitaxial material systems, compressive strain can cause the Raman peak to move away from the intrinsic peak and towards the high wave number (blue shift), while dislocation defects, quantum confinement effect, and tensile strain make the Raman peak shift from intrinsic position to low wave number (red shift). The blue shift of the Ge–Ge peak in samples Ge_2.0_ and Ge_2.4_ is caused by the compressive strain accumulated in the Ge film as described in Eq. (1). Taking into account the effect of atomic interdiffusion based on Eq. (1), the accumulated strain can be written as:

7 $$ E_{\varepsilon } = 0.003528\mu_{e} \cdot \frac{1 + v}{{1 - v}}h \cdot \left( {1 - x} \right)^{2} $$

where ${\mu }_{e}$ and $v$ represent the shear strain and Poisson ratio of Ge film respectively, *h* is the thickness of Ge film, and *x* is the percentage content of Si mixed into Ge film. From Eq. (7), when the deposition thickness *h* of Ge rises to 3.4 nm, the corresponding compressive strain does not necessarily grow in number due to the increased coefficient *x* caused by the Ge-Si mixing, and the high-density and small-sized QDs are formed in Ge_3.4_, which is the evidence that the compressive strain is released following the SK growth mode. From the sample Ge_2.4_ to Ge_3.4_, the huge difference in the morphology of the surface QDs is the main reason that causes their Raman peak positions from blue shift to change into red shift, i.e., the quantum confinement effect plays a dominant role in the red shift in Ge_3.4_. In Ge_4.4_, the quantum confinement effect is greatly weakened because of its too-low QD density and too-large QD size, but its red shift exceeds that of Ge_3.4_, which signifies that the size effect of QDs is not the dominant factor affecting Raman frequency shift in Ge_4.4_, but rather the dislocation defects and the slightly enhanced Ge–Si mixing in the sample. This is consistent with the inference that the typical Ostwald ripening process would generate more dislocation defects.

**Figure 8:**
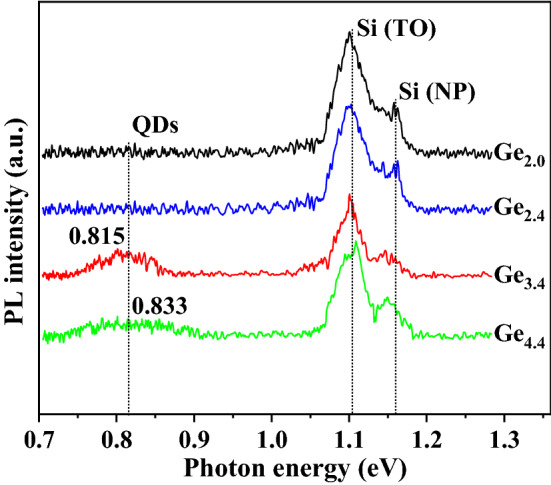
PL spectra of samples Ge_2.0_, Ge_2.4_, Ge_3.4_, and Ge_4.4_ measured at 17 K. The text labels in the figure indicate the signals related to the luminescence originating from the Si transverse optical phonon-assisted recombination (Si (TO), Si non-phonon peak (Si (NP)), and the recombination of excitons in Ge QDs (QDs). The central positions of these PL peaks are marked in the figure.

Figure 8 shows the PL spectra of samples Ge_2.0_, Ge_2.4_, Ge_3.4_, and Ge_4.4_. As shown in the figure, the strong peak centered around 1.100 eV is the Si transverse optical (TO) phonon assisted recombination. The peak located at higher energy than Si TO peak arises from Si no-phonon (NP) recombination. The broad peaks at lower energies (0.815 and 0.833 eV) labeled “QDs” are attributed to the recombination of excitons within the Ge QDs^45^. Compared with the sample Ge_4.4_, the luminescence peak of QDs in Ge_3.4_ exhibits a greater intensity, which is due to the higher density of the QDs on the surface of the sample, and thus more recombination of luminescent excitons. The better size uniformity and spatial distribution uniformity of QDs give rise to the narrowing of the peak in Ge_3.4_. The samples Ge_2.0_ and Ge_2.4_ are difficult to display obvious luminescence information of Ge because their too-thin Ge layer and rare QDs cannot capture enough photons of excitation light.

According to the quantum confinement model^44,46^, assuming that the QDs are standard spheres with a diameter of *d*, then the effective energy gap of these QD materials (*E*_*QD*_) can be expressed as:

8 $$ E_{QD} = E_{g(bulk)} + \frac{{2\hbar^{2} \pi^{2} }}{{\mu d^{2} }} - 3.572\frac{{e^{2} }}{\varepsilon d} - 0.248E_{RY}^{ * } $$

where ${E}_{g(bulk)}$ represents the energy gap of bulk material, *µ* the reduced mass of electrons and holes, and *e* the amount of electron charge, *ɛ* the dielectric constant of vacuum, and ${E}_{RY}^{*}$ the effective Rydberg constant. Equation (8) well describes the dependence between the blue shift of the QD luminescence peak relative to that of bulk material and the QD size, that is, the smaller the average size of QDs, the greater the blue shift of the corresponding luminescence peak. The luminescence peaks of samples Ge_3.4_ and Ge_4.4_ display large blue shifts relative to the intrinsic peak position of 0.738 eV of bulk Ge material, however, contrary to the description of Eq. (8), the central peak position of sample Ge_4.4_ with large-size QDs is slightly higher than that of sample Ge_3.4_ with tiny QDs. This means that in sample Ge_4.4_, in addition to the relatively weak quantum size effect (its QDs are too large in size and too small in density), there are more important dominant factors causing the blue shift of the peak position, which are specifically analyzed as follows: First, there is a larger content of Si components in the QDs of Ge_4.4_. Since the energy gap of Si is higher than that of Ge, the effective energy gap of the alloy structure in QDs increases with the proportion of Si. Second, there are more dislocation defects in the Ge QDs in sample Ge_4.4_, thereby releasing relatively more strain energy accumulated in Ge, the relaxation of the strain raises the effective energy gap of the material. These results and inferences are mutually verified with those obtained from the Raman test of Fig. 7, and through comparison and analysis, it can be predicted that the QD sample Ge_3.4_ with excellent morphology and structure has high application potential in optoelectronic devices, and its growth parameters can be used as the basis for further optimization of higher quality QDs for future devices.

## Conclusions

Based on the characteristics of magnetron sputtering technology, the deposition rates and the crystallization temperatures of Ge and Si, and the epitaxial thickness of the Ge layer suitable for the growth of Ge/Si QDs were explored. The growth and evolution mechanism of QDs prepared by magnetron sputtering at low Ge deposition (1.2–4.4 nm) was further studied. The results showed that the QD evolution conformed to the SK growth mode, however, at a growth temperature of 550 °C, the thickness of the Ge layer (~ 11–14 ML) required for the formation of QDs was much thicker than that of conventional methods such as MBE, CVD, SPE and so on (~ 3–6 ML), which was caused by the non-ideal crystallization and poor flatness of the film caused by insufficient migration of atoms at too high rates. The variation trend of the density and size of QDs with the Ge thickness was similar to that of the conventional methods, but there were great differences in the critical size (volume, diameter, aspect ratio) of the shape transformation of QDs and the existing reports, which was caused by the difference of Ge-Si mixing and dislocation defects between this technology and the conventional methods. In addition, the interpretation of the frequency shift and change of each peak in the Raman and PL spectrum was consistent with the inference obtained from the morphological analysis of QDs, and in these test results, the sample Ge_3.4_ with excellent QD morphology and structure showed relatively optimal photoelectric performance. Our research is a new attempt to adopt the rapid growth method for Ge/Si QDs. The corresponding growth mechanisms and laws can play a beneficial guiding role in realizing the controllable growth of Ge/Si QDs at high deposition rates and have important theoretical foundation significance for promoting the industrial growth and application of QDs in the future.

## Data Availability

The datasets generated during and analyzed during the current study are available from the corresponding author on reasonable request.
